# Supplementary material for: Resolving the full spectrum of human genome variation using Linked-Reads
Source: Genome Res. 2019 Apr;29(4):635–45. doi: 10.1101/gr.234443.118 (PMC6442396; doi:10.1101/gr.234443.118)
Supplement: Supplemental Material [file supp_gr.234443.118_Supplemental_longranger-2.2.zip › longranger-2.2.tar.bz2!/lr2.2-public/lib/stan/cmdstan/stan/doc/api/html/namespacemembers_i.html]

Stan: Namespace Members


|  |  |
| --- | --- |
|  | Stan  2.5.0  probability, sampling & optimization |


- Main Page
- Related Pages
- Namespaces
- Classes
- Files

- Namespace List
- Namespace Members

- All
- Functions
- Variables
- Typedefs
- Enumerations
- Enumerator

- a
- b
- c
- d
- e
- f
- g
- h
- i
- j
- l
- m
- n
- o
- p
- q
- r
- s
- t
- u
- v
- w


All Classes Namespaces Files Functions Variables Typedefs Enumerations Enumerator Friends Macros Pages

Here is a list of all namespace members with links to the namespace documentation for each member:

### - i -

- ibeta()
  : stan::agrad
  , stan::math
- identity\_constrain()
  : stan::prob
- identity\_free()
  : stan::prob
- if\_else()
  : stan::agrad
  , stan::math
- ILL\_FORMED\_T
  : stan::gm
- INDENT()
  : stan::gm
- INDENT2()
  : stan::gm
- INDENT3()
  : stan::gm
- infer\_type\_indexing()
  : stan::gm
- INFTY
  : stan::math
- init\_adapt()
  : stan::common
- init\_nuts()
  : stan::common
- init\_static\_hmc()
  : stan::common
- init\_windowed\_adapt()
  : stan::common
- initialize()
  : stan::math
- initialize\_state()
  : stan::common
- initialize\_state\_random()
  : stan::common
- initialize\_state\_source()
  : stan::common
- initialize\_state\_zero()
  : stan::common
- initialize\_variable()
  : stan::agrad
- input\_iterator\_t
  : stan::gm
- int\_argument
  : stan::gm
- int\_step()
  : stan::math
- INT\_T
  : stan::gm
- integrate\_ode()
  : stan::math
- inv()
  : stan::agrad
  , stan::math
- inv\_chi\_square\_ccdf\_log()
  : stan::prob
- inv\_chi\_square\_cdf()
  : stan::prob
- inv\_chi\_square\_cdf\_log()
  : stan::prob
- inv\_chi\_square\_log()
  : stan::prob
- inv\_chi\_square\_rng()
  : stan::prob
- inv\_cloglog()
  : stan::agrad
  , stan::math
- inv\_gamma\_ccdf\_log()
  : stan::prob
- inv\_gamma\_cdf()
  : stan::prob
- inv\_gamma\_cdf\_log()
  : stan::prob
- inv\_gamma\_log()
  : stan::prob
- inv\_gamma\_rng()
  : stan::prob
- inv\_logit()
  : stan::agrad
  , stan::math
- inv\_sqrt()
  : stan::agrad
  , stan::math
- INV\_SQRT\_2
  : stan::math
- INV\_SQRT\_TWO\_PI
  : stan::math
- inv\_square()
  : stan::agrad
  , stan::math
- inv\_wishart\_log()
  : stan::prob
- inv\_wishart\_rng()
  : stan::prob
- inverse()
  : stan::agrad
  , stan::math
- inverse\_softmax()
  : stan::math
- inverse\_spd()
  : stan::math
- is\_aligned()
  : stan::memory
- is\_assignable()
  : stan::gm
- is\_inf()
  : stan::agrad
  , stan::math
- is\_linear\_function()
  : stan::gm
- is\_nan()
  : stan::agrad
  , stan::math
- is\_nil()
  : stan::gm
- is\_nonempty()
  : stan::gm
- is\_space()
  : stan::gm
- is\_uninitialized()
  : stan::agrad
  , stan::math
- is\_user\_defined()
  : stan::gm
- is\_user\_defined\_prob\_function()
  : stan::gm
- isfinite()
  : boost::math
- isinf()
  : boost::math
  , std
- isnan()
  : boost::math
  , std
- isnormal()
  : boost::math


---

[ Stan Home Page ]

*© 2011–2014,
Stan Development Team.*
